# The Effect of Copper Nanoparticles on Liver Metabolism Depends on the Type of Dietary Fiber

**DOI:** 10.3390/nu16213645

**Published:** 2024-10-26

**Authors:** Aleksandra Marzec, Bartosz Fotschki, Dorota Napiórkowska, Joanna Fotschki, Ewelina Cholewińska, Piotr Listos, Jerzy Juśkiewicz, Katarzyna Ognik

**Affiliations:** 1 Department of Biochemistry and Toxicology, Faculty of Animal Sciences and Bioeconomy, University of Life Sciences in Lublin, Akademicka 13, 20-950 Lublin, Poland; aleksandra.marzec@up.lublin.pl (A.M.); ewelina.cholewinska@up.lublin.pl (E.C.); katarzyna.ognik@up.lublin.pl (K.O.); 2 Division of Food Science, Institute of Animal Reproduction and Food Research, Polish Academy of Sciences, Tuwima 10, 10-748 Olsztyn, Poland; d.napiorkowska@pan.olsztyn.pl (D.N.); j.fotschki@pan.olsztyn.pl (J.F.); j.juskiewicz@pan.olsztyn.pl (J.J.); 3 Sub-Department of Pathomorphology and Forensic Medicine, Faculty of Veterinary Medicine, University of Life Sciences in Lublin, Głęboka 30, 20-612 Lublin, Poland; piotr.listos@up.lublin.pl

**Keywords:** copper nanoparticles, psyllium, dietary fiber, triglycerides, liver metabolism, aminotransferases

## Abstract

Background/Objectives: A diet enriched with copper nanoparticles (CuNPs) exhibits a wide range of effects on liver metabolism, both positive and negative. Dietary fibers are the key components that may affect the absorption of minerals, including copper, and change their impact on organisms. Methods: Therefore, this study investigated whether and how supplementation with different sources of dietary fiber (cellulose, pectin, inulin, and psyllium) affects the function of CuNPs in the liver of male Wistar rats. Results: The results showed that CuNPs at different doses had varying effects on lipid metabolism and inflammation in the liver. Specifically, higher doses of CuNPs were associated with increased lipid accumulation and the activation of pro-inflammatory mechanisms. However, combining CuNPs with dietary fibers, such as psyllium and inulin, was beneficial in mitigating the effects of the examined nanoparticles, leading to reduced fat, cholesterol, and triglycerides in the liver. Combining psyllium with CuNPs showed the most substantial effect on liver metabolism and inflammation parameters. Furthermore, hepatic histology analyses showed that adding psyllium to the diet with CuNPs reduces changes associated with fat accumulation and mononuclear cell infiltration. The observed beneficial changes in the liver may have been related to a reduction in the gene expression level of sterol regulatory element-binding protein 1 and peroxisome proliferator-activated receptor gamma and cyclooxygenase-2. Conclusions: In conclusion, enriching the diet with dietary fibers such as psyllium can regulate the action of CuNPs, thereby improving lipid metabolism and reducing inflammation in the liver.

## 1. Introduction

Due to their small size and high surface-to-volume ratio, copper nanoparticles (CuNPs) exhibit unique properties that make them useful in various applications, including antimicrobial agents, electronics, and agriculture [1,2,3]. Another way to use these nano molecules is as an ingredient in food products. These compounds show a wide range of effects on the consumer’s body, including both positive and negative effects [4].

The liver plays a crucial role in processing compounds absorbed from the digestive tract, making CuNPs in the diet a vital component, with the potential to influence liver metabolism [5]. Using CuNPs as a therapeutic agent for liver metabolic disorders presents promising opportunities as well as significant risks. CuNPs have shown potential in treating non-alcoholic fatty liver disease (NAFLD) by scavenging reactive oxygen species (ROS) and reducing oxidative stress, which are critical factors in the progression of liver diseases [6,7]. Specifically, ultrasmall copper-based nanoparticles combined with resveratrol have demonstrated efficacy in targeting liver tissues and treating inflammation in NAFLD, suggesting a novel approach for managing such conditions [6]. Other in vitro studies have shown the potential of CuNPs as anticancer agents, particularly in targeting colorectal cancer cells [8]. Additionally, the low-dose administration of various inorganic nanoparticles, including CuNPs, has been found to promote lipid degradation and alleviate liver steatosis without causing adverse effects, indicating a potential therapeutic benefit for metabolic regulation [9]. Moreover, the study involving obese individuals with liver steatosis showed that the level of copper in serum played a significant role in predicting the onset of atherosclerosis [10]. However, the hepatotoxicity of CuNPs cannot be overlooked. Studies have shown that high doses of CuNPs can induce significant oxidative stress, inflammation, and histopathological changes in the liver, leading to severe hepatic damage and impaired drug metabolism [11,12]. Furthermore, repeated exposure to CuNPs has been associated with profibrotic changes and immunosuppressive effects [13]. Recent nutritional studies on mice have shown that copper deficiency is strongly associated with metabolic dysfunction-associated steatotic liver disease (MASLD), and CuNPs can exacerbate this condition by disrupting copper metabolism and inducing oxidative stress [14]. The size of the nanoparticles also plays a crucial role, with smaller nanoparticles showing reversible toxicity and better clearance compared to larger ones, which tend to accumulate in the liver and cause prolonged damage [15].

One effective method to modulate the absorption of CuNPs and their impact on the body might be to enhance the diet with fiber. This dietary component can impact the bioavailability of CuNPs through various mechanisms, including mineral binding, fermentation, and interaction with other dietary components. Dietary fibers can bind minerals and form complexes that are difficult to absorb in the intestinal lumen, potentially decreasing mineral bioavailability [16]. However, the type of dietary fiber plays a crucial role in this interaction. For instance, inulin has been shown to have a beneficial effect on DNA repair mechanisms. At the same time, pectin can inhibit inflammatory processes in the small intestine, thereby protecting against the oxidative effects of CuNPs [17]. The physicochemical properties of fibers, such as solubility and fermentability, also influence their interaction with minerals. Soluble fibers are rapidly fermented, which can liberate bound minerals and promote colonic absorption, potentially offsetting any negative effects on mineral bioavailability [18]. Additionally, the interaction between dietary fibers and copper ions can be complex, as demonstrated by the use of Electron Paramagnetic Resonance (EPR) techniques to study barley β-glucan and copper ions, revealing weak interactions under physiological conditions [19].

Previous nutritional research has shown the protective role of dietary fiber supplementation against the potentially harmful oxidative effects of CuNPs in the small intestine [17]. Furthermore, the dietary combination with dietary fiber affects the absorption of CuNPs from the gastrointestinal tract, thus modulating their levels in the liver [20]. However, there is no information on how the type of dietary fiber modulates the effect of CuNPs on liver function. Therefore, the main objective of this study was to evaluate the dosage of CuPNs and dietary combinations of four different sources of dietary fiber (cellulose, pectin, inulin, and psyllium) with CuPNs on the metabolism and inflammation in the liver of rats. As far as animal models are concerned, there is considerable similarity between rodent and human internal organs, including liver functioning. Although in vivo data on metal nanoparticles obtained from rats may not be entirely transferred to humans, it must be stressed that such new information pushes our knowledge forward.

## 2. Materials and Methods

The protocol containing the research questions, experimental schema with the in vivo study design, and analysis plan was submitted to the reviewers of the National Science Center (Kraków, Poland) for evaluation, and was subsequently approved and funded.

### 2.1. Dietary Fibres and Copper Nanoparticles

The copper nanoparticles (99.9% purity) were acquired from SkySpring Nanomaterials, Inc. located in Houston, TX, USA. The detailed physicochemical properties were described by Cholewińska et al. (2023) [17]. For the control dietary sources, CuCO_3_ was purchased from Merck KGaA in Darmstadt, Germany, and α-cellulose from Sigma in Poznań, Poland. Experimental dietary fiber sources such as pectin (PectinE 440(I) from Brouwland in Beverlo, Belgium), inulin (Frutafit Tex from Sensus in Roosendaal, the Netherlands), and psyllium (Psyllium husk powder from NaturaleBio in Rome, Italy) were also used to design the experimental diets.

### 2.2. In Vivo Experiment

A total of 100 nine-week-old healthy outbred male Wistar rats (Cmdb:Wi) were part of the research study. The animals were originated from the Medical University of Bialystok, Poland. After a two-week acclimatization period, they were randomly divided into ten groups, with each group consisting of ten rats. Random numbers were created using Microsoft Excel’s standard = RAND() function (version 15.0.5589.1000). The rats were housed individually in metabolic cages in a carefully controlled environment. The environment included a 12 h light–dark cycle, a temperature maintained at 21 ± 1 °C, a relative humidity ranging from 50% to 70%, and 20 air changes per hour. Throughout a six-week period, each group was given tap water and a modified version of the semipurified casein diet, as recommended for laboratory rodents by the American Institute of Nutrition. The experimental diets in this study consisted of two levels of copper nanoparticles (CuNPs); the recommended level and the double that level were 6.5 and 13 mg/kg, respectively. These levels of CuNPs were paired with various types of dietary fiber. The control diet included a mineral mixture containing standard and high levels of CuCO_3_ (6.5 and 13 mg/kg diet). In contrast, the diets with CuNPs utilized a mineral mixture without CuCO_3_. The control dietary fiber, α-cellulose, was incorporated at 8% of the diet, while the experimental fibers, including inulin with a prebiotic effect, psyllium with a bulking effect, and pectin with a viscous effect, were added at 6% of the diet in place of cellulose. The CuNPs were administered in oil to prevent oxidation, and the experimental diet was kept in the freezer (−70 °C) before administration. The diets provided to the rats throughout the entire experimental period consisted of a detailed composition, which can be found in Table 1. The animals were given unrestricted access to these diets. All animal procedures strictly followed the guidelines outlined in the European Union Directive (2010/63/EU) for animal experiments. Furthermore, the experiment received approval from the local Institutional Animal Care and Use Committee under Permission No. 19/2021 (17 March 2021) in Olsztyn, Poland. The study was conducted in accordance with the ARRIVE guidelines, and all possible measures were taken to minimize the suffering of the animals used in the experiment. During the period of experimental feeding, in the event of adverse effects related to humane endpoints, i.e., cessation of diet intake for more than 2 days, making specific sounds as a pain signal for more than 1 h, the appearance of neurological symptoms (e.g., ataxia, impairment in maintaining a favorable body position), and the presence of blood in the feces for more than 1 day, a veterinarian (employed for these purposes at the institute) could make a decision of humane euthanasia, using the method of gradually filling the chamber of the animal with carbon dioxide or the method of dislodging the cervical vertebrae of a previously sedated animal. To the best of our knowledge, based on literature and our previous experiments, the above symptoms should not be related to the experimental factors (fiber, nanoparticles) and there is only a minimal risk of their occurrence.

### 2.3. Collection of Biological Material and Analytical Procedures

None of the animals were excluded from the experiment. The project manager was the only person who was aware of the animals’ allocation to a particular study group. None of the analysis contractors were acquainted with the treatment animal allocation. During the experiment, the rats were monitored for feed intake and body weight (data published in Cholewińska et al. 2023) [17]. After a period of 6 weeks on the experimental diet, the rats underwent anesthesia with a combination of ketamine and xylazine (100 mg and 10 mg/kg BW, respectively). Subsequently, blood samples were collected from the vena cava into heparinized tubes and then centrifuged at a low speed for 10 min at 350× *g* and 4 °C. The resulting plasma samples were stored at −70 °C until analysis. Following this, the liver was extracted, weighed, and rapidly frozen using liquid nitrogen. Lipids were then extracted from the liver using the Folch et al. (1957) [21] method. A total of 0.2 g of tissue was used for extraction. The extraction process involved using a mixture of chloroform and methanol in a 2/1 ratio. A total of 4 mL of the mixture was added to the tissue and then homogenized. After centrifugation, the chloroform layer was collected, and the solvent was evaporated under nitrogen. The resulting sample was then used for further determinations. The concentrations of liver cholesterol and triglycerides were determined using spectrophotometric techniques with commercial kits (Cholesterol DST, Triglycerides DST, Alpha Diagnostics, Ltd., San Antonio, TX, USA). The sample size was determined for each analysis based on previous research.

### 2.4. Blood Plasma Parameters of Metabolism and Inflammation

The plasma concentration of cholesterol (total and its HDL), triglycerides, creatinine, uric acid, urea, glucose, and the plasma activities of aspartate transaminase (AST) and alanine transaminase (ALT) were determined using an automatic biochemical analyzer (Pentra C200, Horiba, Tokyo, Japan). The atherogenic index of plasma was calculated according to the following formula: log(TG/HDL). To measure the concentration of insulin, glucagon, and ghrelin in the plasma, validated rat ELISA kits were used (Shanghai Qayee Biotechnology Co. Ltd., Shanghai, China).

### 2.5. Liver Gene Expression

Firstly, RNA was extracted from the liver using a TRI Reagent solution (Thermo Fisher Scientific, Waltham, MA, USA), as per the manufacturer’s instructions. *β-actin* was chosen as the reference gene. The mRNA expression levels of peroxisome proliferator-activated receptor gamma (*ppar-γ*, Cat# Rn00440945_m1), peroxisome proliferator-activated receptor alpha (*ppar-α*, Cat# Rn00566193_m1), sterol regulatory element-binding protein 1 (*srebp1c*, Cat# Rn01495769_m1), and cyclooxygenase-2 (*cox-2*, Cat# Rn01483828_m1) were measured using single tube TaqMan^®^ Gene Expression Assays (Life Technologies, Carlsbad, CA, USA). Amplification was carried out using a 7900HT Fast Real-Time PCR System under the following conditions: initial denaturation for 10 min at 95 °C, followed by 40 cycles of 15 s at 95 °C and 1 min at 60 °C. Finally, the mRNA expression levels of the selected genes were normalized to *β-actin* (Cat# Rn00667869_m1).

### 2.6. Liver Histopathology

Histopathological examinations, including hematoxylin eosin staining of liver samples from rats, were conducted following the procedure described in our previous study [22].

### 2.7. Statistical Analysis

The differences among treatment groups were determined using STATISTICA software, version 12.0 (StatSoft Corp., Krakow, Poland). A two-way analysis of variance (ANOVA) was used to evaluate the effects of two main factors, the dosage of copper nanoparticles (CuNPs) (low dose, 6.5 mg/kg and high dose, 13 mg/kg) and the type of dietary fiber (cellulose, pectin, inulin, and psyllium). Following the ANOVA, Duncan’s multiple range test was conducted. The data were checked for normality with the aid of the Shapiro–Wilk test. Furthermore, to compare each experimental group fed the low dose of CuNPs with the control group (fed a diet with 6.5 mg/kg Cu from CuCO_3_ and containing cellulose as the primary dietary fiber source), a *t*-test was employed. Similarly, the *t*-test was used to compare the experimental groups fed diets with the high dose of CuNPs with the control group, CH, in which rats were fed a diet with 13 mg/kg Cu from CuCO_3_ and containing cellulose as the primary dietary fiber source. Differences with a significance level of *p* ≤ 0.05 were considered to be statistically significant.

## 3. Results

The basic parameters of growth and dietary intake shown in previous work [17] indicate that the experimental diets had no effect on the final weight and body weight gain in rats, while dietary intake significantly decreased when inulin and psyllium were added to the diets with CuNPs. The relative weight of the liver was the highest in rats fed diets with inulin (*p* < 0.05 vs. the treatment with psyllium), regardless of the CuNPs dose (Table 2). The D × F interaction showed the highest fat content was observed in the hepatic tissue in the CHH and PN groups (*p* < 0.05 vs. all other groups), while the lowest fat content was noted in the SNH rats (*p* < 0.05 vs. remaining groups except SN and JN). Irrespective of the nanoparticle dose, psyllium treatment decreased hepatic total cholesterol and triglycerides concentration, as well as the activity of plasma ALT, as compared to other treatments with cellulose, pectin, or inulin. Additionally, we observed reduced ALT activity in the pectin treatment in comparison to the cellulose one (*p* < 0.05). The CuNPs dose by fiber interaction showed the highest plasma AST activity occurred in the CNH and PNH rats (*p* < 0.05 vs. remaining groups except CN), while the lowest AST was noted in the JN and JNH groups (*p* < 0.05 vs. CN, CNH, PNH).

The *t*-test revealed a diminished relative liver weight in the PN and SN rats compared to the C control (*p* < 0.05). Hepatic fat concentration was significantly enhanced in the CNH and PN groups vs. their respective controls, i.e., the CH and C, respectively (*t*-test; *p* < 0.05). In the remaining groups fed diets with copper nanoparticles, except PNH, the liver fat concentration was decreased in comparison to the respective controls fed diets with CuCO_3_ (*t*-test; *p* < 0.05). As compared to the respective controls, the hepatic total cholesterol was diminished in all experimental groups with CuNPs, except CNH and JN. In the case of hepatic triglycerides, such decreases were noted in the CN, JN, JHNH, SN, and SNH rats (*p* < 0.05; *t*-test). The *t*-test showed significantly enhanced blood plasma AST activity in the CN (vs. C), CNH (vs. CH), and PNH (vs. CH) groups. The plasma ALT activity was lower in SNH rats than CH ones (*t*-test; *p* < 0.05).

The two-way ANOVA showed that irrespective of the CuNPs dose, the treatments with functional fiber, i.e., pectin, inulin, and psyllium, caused a significant increase in the plasma HDL concentration, thus decreasing the TC/HDL ratio compared to the cellulose treatment (Table 3). The lowest AIP index value was noted in the psyllium treatment, regardless of the CuNPs dose (*p* < 0.05 vs. C and P treatments). Additionally, the AIP noted in the inulin treatment was lower than in the C one (*p* < 0.05). The *t*-test showed that in comparison to a respective control, the SNH rats had decreased creatinine concentrations, and the PNH and SN animals had decreased uric acid levels in their blood plasma. As compared to the C control, plasma urea was significantly enhanced in the JN group (*t*-test). The plasma HDL concentration was enhanced in the PN and SN rats (vs. control C; *t*-test), as well as in the JNH and SNH animals (vs. control CH; *t*-test). The TC/HDL ratio decreased in the PN, JN, JNH, SN, and SNH rats in comparison to their respective controls (*p* < 0.05; *t*-test). The AIP index decreased in rats JNH vs. CH (*p* < 0.05; *t*-test).

A significant CuNPs by fiber interaction showed that insulin concentration in the plasma of rats fed diets PN, PNH, JN, and SNH was enhanced as compared to JNH rats (*p* < 0.05; Table 4). The highest and lowest plasma ghrelin concentration was noted in the JNH and SNH groups, respectively (in both cases *p* < 0.05 vs. remaining groups). The highest glucagon concentration in the plasma followed feeding with diet CN (*p* < 0.05 vs. all other groups, except CNH), while the lowest glucagon levels were noted in the PN, JNH, SN, and SNH rats (*p* < 0.05 vs. other groups; see significant D×F interaction). The *t*-test revealed that all groups fed diets with copper nanoparticles were characterized by diminished plasma insulin levels when compared to their respective controls (*p* < 0.05 vs. C or CH). The ghrelin concentration in the plasma was enhanced in JNH rats vs. CH rats, while in the SNH rats, ghrelin levels were lower compared to the CH control (*p* < 0.05; *t*-test). The glucagon content in the plasma was decreased in the groups fed diets with copper nanoparticles, except CN, CNH, and PNH in comparison to the respective controls without CuNPs (*p* < 0.05; *t*-test).

**Figure 1:**
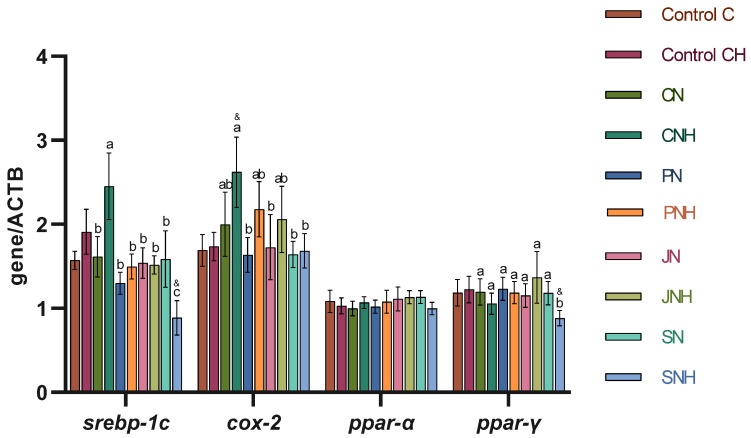
Hepatic mRNA expression (normalized to β-actin) of metabolic and inflammatory factors in rats fed experimental diets (n = 10 per group). Description of the groups same as in Table 2. *cox-2*, cyclooxygenase-2; *ppar-α*, peroxisome proliferator-activated receptor alpha; *ppar-γ*, peroxisome proliferator-activated receptor gamma; *srebp-1c*, sterol regulatory element-binding protein 1. Mean values within a column with different superscript letters differ significantly (*p* < 0.05). Similarly, each experimental group fed CuNPs 13 mg/kg (CNH, PNH, JNH, SNH) was compared with the control CH group using a *t*-test (& indicates a significant difference versus the CH group).

The hepatic *srebp-1c* expression was significantly enhanced in the CNH group, while the lowest expression of *srebp-1c* in the liver was observed in the SNH rats (Figure 1; in both cases *p* < 0.05 vs. all other groups). The CNH rats were also characterized by the highest hepatic *cox-2* expression (*p* < 0.05 vs. PN, JN, SN, SNH). The SNH liver had decreased *ppar-γ* expression as compared to other groups (*p* < 0.05). The *t*-test showed a decrease in *srebp-1c* and *ppar-γ* hepatic expression in SNH rats vs. control CH (*p* < 0.05). The *cox-2* expression was significantly higher in the CNH group than in the CH one (*t*-test). 

**Figure 2:**
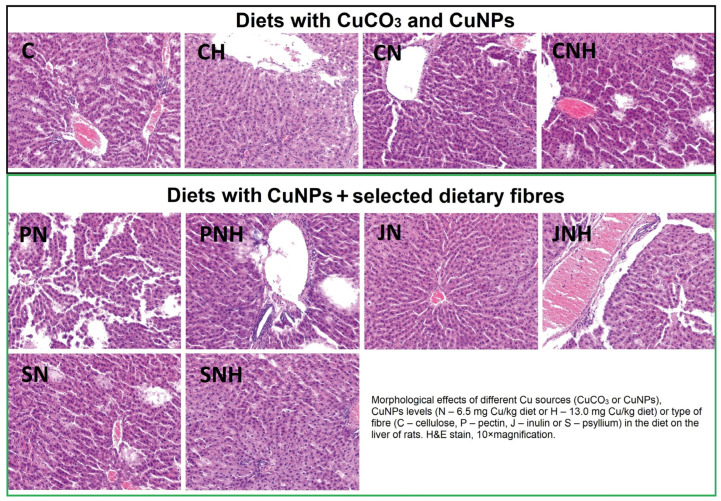
Hepatic histology in rats fed experimental diets. Description of the groups same as in Table 2. C, single foci of fatty degeneration of a physiological nature combined with the presence of small clusters of mononuclear cell infiltration; CH, physiological structure combined with slight congestion of the liver; CN, physiological structure of the liver and the presence of multiple small foci of fatty degeneration; CNH, extensive multiple-multifocal foci of fatty degeneration of the liver; PN, extensive singular fatty degeneration of the liver; PNH, extensive multifocal fatty degeneration of the liver; JN, physiological structure of the liver and presence of multiple small foci of fatty degeneration, a single cluster of mononuclear cell infiltration; JNH, multiple small fatty degeneration of the liver, combined with multiple clusters of mononuclear cell infiltration; SN, presence of multiple small foci of fatty degeneration, a single cluster of mononuclear cell infiltration; SNH, single-focal fatty degeneration of the liver, combined with single clusters of mononuclear cell infiltration.

The histopathological examination revealed minor changes in the liver structure of rats from the C and CH groups (Figure 2). A physiological structure with small clusters of mononuclear cell infiltration and slight congestion of the liver was observed in the CH group. The CN group showed a normal liver structure without any pathological changes. In the SN group, minor histological changes were observed, including minor fatty changes and mononuclear cell infiltration. Additionally, the SNH, JN, and JNH groups, which were administered with inulin and psyllium, exhibited mild fatty degeneration and single clusters of mononuclear cell infiltration. The most severe changes, characterized by extensive fatty degeneration, were observed in the CNH, PN, and PNH groups.

## 4. Discussion

The present experiment involves Wistar rats (Cmdb:Wi) because the rat model is an established host model for nutritional and metabolic studies, including the gastrointestinal response to nutritional interventions and the systemic (metabolic) changes observed with test dietary supplementation. Of course, there are limitations of applying findings from an animal study to humans. These limitations stem from differences in species, fiber, and copper content of the diet, and the inability to test dietary nanoparticles on humans. However, sufficient literature supports the statement that the rat model provides important strengths for the study of human health and disease in relation to human dietary habits and environment. Copper is essential for numerous metabolic activities, including lipid metabolism, redox balance, and iron mobilization, and its homeostasis is crucial for maintaining cellular function [23,24]. Dysregulation of copper levels can lead to oxidative stress, which is implicated in developing many liver metabolic disorders. Recently, there has been much interest in using nanocompounds in food. Due to their size and surface interaction area, these compounds may affect organisms differently than their native form. Indeed, in this study, replacing CuCO_3_ with CuNPs at a dose of 6.5 mg/kg significantly reduced fat, cholesterol, and triglycerides in the liver, while increasing the dose to 13 mg/kg showed the opposite effect. Due to the enhanced interaction of nano Cu with the body, they have a more significant impact, which may be comparable to an excess of copper in the diet. Other studies also showed that excess Cu could induce mitochondrial dysfunction, promoting lipid deposition and lipogenesis [25]. Furthermore, histological analysis confirmed the observed changes regarding the effect of CuNPs dose on hepatic lipid metabolism. Numerous multifocal foci of lipid degeneration were noted in the group with an increased dose of CuNPs. Irrespective of the dose, CuNPs increased plasma aspartate aminotransferase activity, which may indicate the activation of inflammatory mechanisms in the liver. These changes were most likely related to the increased expression levels of *srebp-1c* and *cox-2* in groups with CuNPs, which are involved in lipid accumulation mechanisms and liver inflammation development [26,27]. Srebp-1c is intricately linked to insulin signaling and plays a pivotal role in lipid and glucose metabolism. Insulin stimulates the transcription of the *srebp-1c* gene, activating the transcription of the genes necessary for fatty acid synthesis, thereby promoting de novo lipogenesis in the liver [26,28]. Interestingly, in this study, blood insulin levels decreased significantly, while glucose levels did not change when the dietary source of Cu was replaced with CuNPs. Other studies showed that srebp-1c in the liver represses insulin receptor substrate-2 (IRS-2) transcription by displacing forkhead proteins from the insulin response element on the IRS-2 promoter, contributing to hepatic insulin resistance [29]. This indicates that an increased dose of CuNPs can significantly interfere with the regulation of glucose and lipid metabolism in the liver. This may disrupt the mechanism linking the interaction of *srebp-1c* and insulin, thus increasing liver fat accumulation and developing inflammation.

Our previous nutritional study with CuNPs suggests that adding dietary fiber to rats’ diets decreases Cu intake, affecting Cu bioavailability [20]. Based on these results, it was assumed that combining CuNPs with dietary fibers may regulate the intensity of the nanocompound’s action on hepatic metabolism. Indeed, this study confirmed that the beneficial effects of combining CuNPs with dietary fiber depend on the type of fiber. The combination of psyllium and CuNPs in the diet, regardless of dose, was most beneficial in reducing fat, total cholesterol, and triglycerides in the liver. In addition, a comparable effect was observed when inulin was combined with an increased dose of CuNPs in the diet. While inulin and psyllium offer significant health benefits, their nutritional and functional properties differ. Inulin is primarily valued for its prebiotic effects and its role in enhancing mineral absorption, metabolic health, and reducing inflammation [30]. In contrast, psyllium is prized for its ability to form a gel-like substance in the gut that helps slow digestion, manage blood sugar levels, and lower cholesterol [31]. Therefore, adding fibers like psyllium and inulin to the diet could be an explanation for the mitigation of the CuNPs effect by reducing the activity of aminotransferases in the blood. Additionally, higher blood HDL levels and lower atherogenicity factor values were observed in the groups with CuNPs supplemented with psyllium and inulin. The same was observed in the histological analysis, where adding inulin and psyllium to a diet with a higher dose of CuNPs reduced the foci of fatty degeneration of the liver. The beneficial effects of combining psyllium with CuNPs on lipid metabolism and activating pro-inflammatory mechanisms in the liver are most likely related to the observed downregulation of *srebp1c*, *ppar-γ*, and *cox-2* expression levels. These molecule indicators are involved in many mechanisms regulating lipids, glucose metabolism, and inflammation in the liver [26,32]. Particularly interesting are the changes in *ppar-γ* expression. Activation of this receptor in the liver can lead to the increased storage of fatty acids in the form of triglycerides, which is a protective mechanism against lipotoxicity, preventing the accumulation of free fatty acids that can cause cellular damage [33,34]. This mechanism may explain why liver triglyceride levels were reduced in the group with psyllium.

Moreover, *ppar-γ* plays a crucial role in glucose metabolism. It enhances insulin sensitivity by modulating the expression of genes involved in glucose uptake and utilization. It is essential in developing NAFLD and type 2 diabetes, where insulin resistance is a standard disorder. By improving insulin sensitivity, *ppar-γ* activation can help reduce hepatic glucose production and improve overall glucose homeostasis [35,36]. Interestingly, when the examined fibers were added to the diets, CuPNs’ insulin-lowering effect with unchanged blood glucose levels was also observed. However, the examined dietary fibers reduced glucagon levels. Glucagon is produced by the alpha cells of the pancreas and serves to increase blood glucose levels, acting as a counter-regulatory hormone to insulin [37]. Consequently, the natural mechanism for lowering blood insulin levels involves a decrease in glucagon secretion. This mechanism was disrupted when CuNPs were added to the diet. However, adding dietary fibers (specifically psyllium) reversed the action of CuNPs and lowered glucagon levels.

Another mechanism that can indirectly influence blood insulin levels is related to ghrelin secretion. Ghrelin has been shown to inhibit insulin secretion, which can lead to increased blood glucose levels and potentially contribute to insulin resistance over time [38]. When comparing the dietary fibers tested, psyllium showed the most significant reduction in blood ghrelin levels. However, inulin increases the level of this hormone. This effect may also explain the lowest insulin level observed in the group with CuNPs and inulin. One of the mechanisms regulating ghrelin secretion is associated with the feeling of satiety. Therefore, the characteristic gelling effect of psyllium [31], which can make it easier to feel full, may also reduce the secretion of this hormone. The effect of a decrease in ghrelin levels matches the reduced dietary intake in the psyllium and CuNPs group described in an earlier paper [17]. For inulin, the mechanism of increasing ghrelin levels in blood is probably associated with its prebiotic properties and the production of short-chain fatty acids (SCFAs). The fermentation of inulin by gut bacteria increases the production of SCFAs, which can enhance the secretion of ghrelin by stimulating the enteroendocrine cells in the stomach and the small intestine. Moreover, the increase in SCFAs can also affect the expression of genes involved in ghrelin production [39,40,41].

## 5. Conclusions

In conclusion, the gathered data show that CuNPs can significantly impact hepatic lipid metabolism and inflammatory mechanisms in the liver. The study revealed that replacing CuCO_3_ with CuNPs at different doses had distinct, unfavorable effects on liver fat, cholesterol, and triglyceride levels. High doses of CuNPs induced lipid degeneration and activated inflammatory mechanisms, as indicated by the increased plasma aspartate aminotransferase activity and the elevated expression levels of specific genes associated with lipid accumulation and liver inflammation. Interestingly, the study also demonstrated that combining CuNPs with dietary fibers, such as psyllium and inulin, modulates the effects of CuNPs on hepatic metabolism. Combining CuNPs with these dietary fibers showed beneficial effects in reducing fat, cholesterol, and triglycerides, while also decreasing the activity of aminotransferases in the blood. Moreover, it was observed that adding psyllium and inulin resulted in higher blood HDL levels and lower liver fatty degeneration foci when combined with CuNPs. These findings underscore the intricate relationship between CuNPs, dietary fibers, and hepatic metabolism. The addition of psyllium to the diet with CuNPs downregulated specific gene expression levels, such as *srebp-1c, ppar-γ*, and *cox-2*, responsible for the regulation of lipids, glucose metabolism, and inflammatory pathways in the liver. The study highlights the potential of dietary fibers in reducing the negative effects of CuNPs on liver function, opening the door for further research into the combined effects of nanocompounds and dietary interventions on metabolic health. However, it is important to note that the potential toxicity of CuNPs still poses a significant barrier to clinical translation.

## Figures and Tables

**Table 1:** The experimental diets given to the rats for six weeks.

	C	CH	CN	CNH	PN	PNH	JN	JNH	SN	SNH
					%					
Casein ^1^	14.8	14.8	14.8	14.8	14.8	14.8	14.8	14.8	14.8	14.8
DL-methionine	0.2	0.2	0.2	0.2	0.2	0.2	0.2	0.2	0.2	0.2
Cellulose ^2^	8.0	8.0	8.0	8.0	2.0	2.0	2.0	2.0	2.0	2.0
Pectin					6	6				
Inulin							6	6		
Psyllium									6	6
Other components ^3^	77.0	77.0	77.0	77.0	77.0	77.0	77.0	77.0	77.0	77.0
Calculation:										
Cu from, mg/kg										
CuCO_3_	6.5	13	0	0	0	0	0	0	0	0
CuNPs	0	0	6.5	13	6.5	13	6.5	13	6.5	13

C, control diet with a standard Cu content in the mineral mix and 8% of cellulose as a dietary fiber source; CH, control diet with an enhanced Cu content in the mineral mix and 8% of cellulose as a dietary fiber; CN, diet with a standard CuNPs content in the mineral mix and 8% of cellulose as a dietary fiber source; CNH, diet with enhanced CuNPs content in the mineral mix and 8% of cellulose as a dietary fiber; PN, diet with a standard CuNPs content in the mineral mix and 2% of cellulose and 6% of pectin as a dietary fiber source; PNH, diet with enhanced CuNPs content in the mineral mix and 2% of cellulose and 6% of pectin as a dietary fiber source; JN, diet with a standard CuNPs content in the mineral mix and 2% of cellulose and 6% of inulin as a dietary fiber source; JNH, diet with enhanced CuNPs content in the mineral mix and 2% of cellulose and 6% of inulin as a dietary fiber source; SN, diet with a standard CuNPs content in the mineral mix and 2% of cellulose and 6% of psyllium as a dietary fiber source; SNH, diet with enhanced CuNPs content in the mineral mix and 2% of cellulose and 6% of psyllium as a dietary fiber source. ^1^ Casein preparation: crude protein 89.7%, crude fat 0.3%, ash 2.0%, and water 8.0%. ^2^ α-Cellulose (SIGMA, Poznan, Poland), main source of dietary fiber. ^3^ 0.2% choline chloride, 8.0% rapeseed oil, 64.0% maize starch, 1.0% vitamin mix (AIN-93G-VM), and 3.5% mineral mix were added to each diet. In the experimental treatments involving copper nanoparticles (CuNPs), the mineral mix was intentionally devoid of copper carbonate (CuCO_3_). To ensure the safety of the operator during the preparation of the experimental diets, the CuNPs preparation was incorporated into an emulsion, along with dietary rapeseed oil. This approach, which has been successfully employed in the previous study, helped to mitigate potential risks associated with handling CuNPs and ensured the effective administration of the treatment.

**Table 2:** Liver function indicators and the levels of AST and ALT in the blood plasma of rats that were fed experimental diets (*n* = 10 per group) *.

	Weight	Fat	TC	TG	AST	ALT
	g/100 g BW	%	mg/g	mg/g	U/L	U/L
Control C	2.80 ± 0.04	14.8 ± 0.74	10.9 ± 1.03	13.8 ± 1.08	57.5 ± 1.55	17.6 ± 0.93
Control CH	2.83 ± 0.07	13.8 ± 0.97	10.8 ± 0.68	13.3 ± 0.93	59.6 ± 1.14	19.0 ± 0.99
Two-way ANOVA:						
CN	2.83 ± 0.08	12.2 ± 0.96 ^b#^	6.42 ± 0.58 ^#^	10.8 ± 0.48 ^#^	63.1 ± 0.67 ^ab#^	19.1 ± 0.66
CNH	2.78 ± 0.06	18.8 ± 1.39 ^a&^	8.71 ± 1.04	11.1 ± 1.15	66.7 ± 1.88 ^a&^	21.8 ± 1.10
PN	2.67 ± 0.04 ^#^	18.3 ± 1.13 ^a#^	6.40 ± 0.79 ^#^	11.4 ± 1.07	59.2 ± 1.82 ^bc^	18.2 ± 0.83
PNH	2.73 ± 0.06	12.5 ± 0.81 ^b^	7.11 ± 0.88 ^&^	12.8 ± 0.98	68.1 ± 2.07 ^a&^	18.5 ± 0.45
JN	2.78 ± 0.07	8.26 ± 0.85 ^cd#^	8.25 ± 1.09	10.8 ± 0.98 ^#^	55.7 ± 1.65 ^c^	19.1 ± 0.96
JNH	2.93 ± 0.18	8.49 ± 0.84 ^c&^	7.43 ± 0.47 ^&^	10.4 ± 0.85 ^&^	55.5 ± 1.88 ^c^	19.3 ± 0.93
SN	2.58 ± 0.06 ^#^	7.90 ± 0.57 ^cd#^	3.67 ± 0.23 ^#^	9.11 ± 0.56 ^#^	60.0 ± 1.91 ^bc^	16.6 ± 0.78
SNH	2.65 ± 0.05	5.56 ± 067 ^d&^	2.97 ± 0.55 ^&^	6.87 ± 0.57 ^&^	60.1 ± 1.91 ^bc^	16.4 ± 0.73 ^&^
SEM	0.026	0.511	0.344	0.333	0.654	0.297
CuNPs dose (D)						
L (6.5 mg/kg)	2.72	11.6	6.18	10.5	59.5	18.2
H (13 mg/kg)	2.76	11.4	6.55	10.3	62.3	18.9
*p* value	0.509	0.656	0.487	0.697	0.025	0.243
Fiber type (F)						
C (cellulose)	2.78 ^ab^	15.5	7.56 ^a^	11.0 ^a^	64.9	20.5 ^a^
P (pectin)	2.70 ^ab^	15.4	6.76 ^a^	12.1 ^a^	63.7	18.3 ^b^
J (inulin)	2.85 ^a^	8.38	7.84 ^a^	10.6 ^a^	55.0	19.1 ^ab^
S (psyllium)	2.62 ^b^	6.73	3.32 ^b^	7.99 ^b^	60.0	16.5 ^c^
*p* value	0.041	<0.001	<0.001	<0.001	<0.001	<0.001
Interaction D × F						
*p* value	0.504	<0.001	0.146	0.212	0.022	0.256

* The experimental feeding period involved different dietary treatments: groups C, control diet with a standard Cu content in the mineral mix and 8% of cellulose as a dietary fiber source; group CH, control diet with an enhanced Cu content in the mineral mix and 8% of cellulose as a dietary fiber; group CN, diet with a standard CuNPs content in the mineral mix and 8% of cellulose as a dietary fiber source; group CNH, diet with enhanced CuNPs content in the mineral mix and 8% of cellulose as a dietary fiber; group PN, diet with a standard CuNPs content in the mineral mix and 2% of cellulose and 6% of pectin as a dietary fiber source; group PNH, diet with enhanced CuNPs content in the mineral mix and 2% of cellulose and 6% of pectin as a dietary fiber source; group JN, diet with a standard CuNPs content in the mineral mix and 2% of cellulose and 6% of inulin as a dietary fiber source; group JNH, diet with enhanced CuNPs content in the mineral mix and 2% of cellulose and 6% of inulin as a dietary fiber source; group SN, diet with a standard CuNPs content in the mineral mix and 2% of cellulose and 6% of psyllium as a dietary fiber source; group SNH, diet with enhanced CuNPs content in the mineral mix and 2% of cellulose and 6% of psyllium as a dietary fiber source. Mean values within a column with different superscript letters differ significantly (*p* < 0.05). Differences among the groups (CN, CNH, PN, PNH, JN, JNH, SN, SNH) are indicated with superscripts only if there is a statistically significant interaction D × F (*p* < 0.05). Each experimental group fed CuNPs 6.5 mg/kg (CN, PN, JN, SN) was compared with the control C group using a *t*-test (# indicates a significant difference versus the C group). Similarly, each experimental group fed CuNPs 13 mg/kg (CNH, PNH, JNH, SNH) was compared with the control CH group using a *t*-test (& indicates a significant difference versus the CH group). The results are presented as the mean ± standard error of the mean (SEM). ALT, alanine aminotransferase; AST, aspartate aminotransferase; TC, total cholesterol; TG, triglycerides.

**Table 3:** Blood plasma parameters in rats fed experimental diets (*n* = 10 per group) *.

	Creat.	UA	Urea	HDL	TC	TG	GL	AIP	TC/HDL
	µmol/L	µmol/L	mmol/L	mmol/L	mmol/L	mmol/L	mmol/L		
Control C	23.6 ± 0.95	35.4 ± 1.85	3.99 ± 0.22	0.397 ± 0.026	1.58 ± 0.09	0.927 ± 0.08	12.4 ± 0.63	0.361 ± 0.049	4.04 ± 0.18
Control CH	24.0 ± 0.72	34.2 ± 2.28	4.20 ± 0.09	0.417 ± 0.022	1.69 ± 0.08	1.05 ± 0.07	13.9 ± 0.71	0.399 ± 0.033	4.08 ± 0.13
Two-way ANOVA:									
CN	25.0 ± 1.03	33.4 ± 1.82	4.20 ± 0.11	0.442 ± 0.023	1.75 ± 0.08	1.22 ± 0.12	13.3 ± 0.85	0.427 ± 0.045	4.01 ± 0.16
CNH	23.5 ± 0.71	34.8 ± 2.54	4.35 ± 0.17	0.417 ± 0.030	1.66 ± 0.08	1.01 ± 0.05	13.8 ± 0.57	0.387 ± 0.039	4.06 ± 0.19
PN	22.7 ± 0.95	29.7 ± 2.59	4.28 ± 0.09	0.491 ± 0.022 ^#^	1.64 ± 0.07	1.16 ± 0.08	13.7 ± 0.50	0.367 ± 0.034	3.35 ± 0.08 ^#^
PNH	22.7 ± 0.91	28.2 ± 1.60 ^&^	4.39 ± 0.15	0.467 ± 0.027	1.66 ± 0.08	1.16 ± 0.10	13.7 ± 0.42	0.389 ± 0.041	3.61 ± 0.18
JN	22.5 ± 0.89	30.1 ± 2.56	4.84 ± 0.19 ^#^	0.462 ± 0.031	1.56 ± 0.07	0.901 ± 0.08	13.2 ± 0.76	0.283 ± 0.046	3.46 ± 0.19 ^#^
JNH	23.7 ± 1.00	33.2 ± 3.15	4.57 ± 0.20	0.499 ± 0.019 ^&^	1.61 ± 0.09	0.960 ± 0.05	14.2 ± 0.63	0.283 ± 0.026 ^&^	3.26 ± 0.23 ^&^
SN	23.3 ± 1.63	28.9 ± 1.96 ^#^	4.56 ± 0.33	0.499 ± 0.022 ^#^	1.67 ± 0.09	0.926 ± 0.14	12.4 ± 0.37	0.200 ± 0.100	3.35 ± 0.14 ^#^
SNH	21.8 ± 0.74 ^&^	29.5 ± 2.22	4.28 ± 0.17	0.504 ± 0.019 ^&^	1.73 ± 0.08	1.12 ± 0.12	12.7 ± 0.64	0.326 ± 0.046	3.44 ± 0.10 ^&^
SEM	0.309	0.740	0.060	0.008	0.025	0.030	0.198	0.016	0.059
CuNPs dose (D)									
L (6.5 mg/kg)	23.4	30.5	4.47	0.474	1.66	1.05	13.1	0.319	3.54
H (13 mg/kg)	22.7	31.5	4.42	0.472	1.67	1.06	13.6	0.346	3.60
*p* value	0.382	0.548	0.692	0.932	0.841	0.864	0.294	0.470	0.622
Fibre type (F)									
C (cellulose)	24.2	34.1	4.27	0.430 ^b^	1.70	1.11	13.5	0.407 ^a^	4.04 ^a^
P (pectin)	22.7	29.0	4.33	0.479 ^a^	1.65	1.16	13.7	0.378 ^ab^	3.48 ^b^
J (inulin)	22.8	31.9	4.75	0.481 ^a^	1.59	0.933	13.7	0.284 ^bc^	3.37 ^b^
S (psyllium)	22.5	29.2	4.42	0.502 ^a^	1.70	1.02	12.6	0.263 ^c^	3.40 ^b^
*p* value	0.285	0.105	0.070	0.033	0.511	0.112	0.221	0.017	<0.001
Interaction D × F									
*p* value	0.687	0.766	0.594	0.551	0.719	0.232	0.931	0.435	0.662

* Description same as in Table 2. The results are presented as the mean ± standard error of the mean (SEM). Mean values within a column with different superscript letters differ significantly (*p* < 0.05). Differences among the groups (CN, CNH, PN, PNH, JN, JNH, SN, SNH) are indicated with superscripts only if there is a statistically significant interaction D × F (*p* < 0.05). Each experimental group fed CuNPs 6.5 mg/kg (CN, PN, JN, SN) was compared with the control C group using a *t*-test (# indicates a significant difference versus the C group). Similarly, each experimental group fed CuNPs 13 mg/kg (CNH, PNH, JNH, SNH) was compared with the control CH group using a *t*-test (& indicates a significant difference versus the CH group). TC, total cholesterol (mmol/L); HDL, high density lipoprotein (mmol/L); TG, triglycerides (mmol/L); AIP, atherogenic index of plasma [log(TG/HDL)]; UA, uric acid (µmol/L); Urea, mmol/L; Creat., creatinine (µmol/L); GL, glucose (mmol/L).

**Table 4:** Blood levels of insulin, ghrelin, and glucagon in rats fed experimental diets.(*n* = 10 per group) *.

	Insulin	Ghrelin	Glucagon
	µIU/mL	pg/mL	ng/mL
Control C	16.6 ± 0.71	30.4 ± 1.46	9.99 ± 0.36
Control CH	15.6 ± 0.74	28.7 ± 1.61	8.37 ± 0.58
Two-way ANOVA:			
CN	10.7 ± 0.92 ^ab#^	30.0 ± 1.94 ^b^	9.70 ± 0.92 ^a^
CNH	10.6 ± 0.30 ^ab&^	29.2 ± 1.78 ^b^	9.48 ± 0.51 ^ab^
PN	11.6 ± 0.46 ^a#^	29.5 ± 1.05 ^b^	5.53 ± 0.38 ^d#^
PNH	12.1 ± 0.72 ^a&^	30.6 ± 1.18 ^b^	7.07 ± 0.60 ^c^
JN	12.0 ± 0.50 ^a#^	30.7 ± 2.08 ^b^	8.03 ± 0.31 ^bc#^
JNH	9.71 ± 0.52 ^b&^	35.1 ± 0.64 ^a&^	5.52 ± 0.50 ^d&^
SN	10.9 ± 0.41 ^ab#^	29.9 ± 1.46 ^b^	5.23 ± 0.44 ^d#^
SNH	11.7 ± 0.52 ^a&^	20.4 ± 1.15 ^c&^	4.99 ± 0.33 ^d&^
SEM	0.279	0.568	0.246
CuNPs dose (D)			
L (6.5 mg/kg)	11.3	30.0	7.12
H (13 mg/kg)	11.0	28.8	6.76
*p* value	0.489	0.258	0.342
Fiber type (F)			
C (cellulose)	10.7	29.6	9.59
P (pectin)	11.8	30.0	6.30
J (inulin)	10.8	32.9	6.78
S (psyllium)	11.3	25.2	5.11
*p* value	0.181	<0.001	<0.001
Interaction D × F			
*p* value	0.042	<0.001	0.004

* Description same as in Table 2. The results are presented as the mean ± standard error of the mean (SEM). Mean values within a column with different superscript letters differ significantly (*p* < 0.05). Differences among the groups (CN, CNH, PN, PNH, JN, JNH, SN, SNH) are indicated with superscripts only if there is a statistically significant interaction D × F (*p* < 0.05). Each experimental group fed CuNPs 6.5 mg/kg (CN, PN, JN, SN) was compared with the control C group using a *t*-test (# indicates a significant difference versus the C group). Similarly, each experimental group fed CuNPs 13 mg/kg (CNH, PNH, JNH, SNH) was compared with the control CH group using a *t*-test (& indicates a significant difference versus the CH group).

## Data Availability

The original contributions presented in the study are included in the article, further inquiries can be directed to the corresponding author.
